# Invasive Group A *Streptococcus* Hypervirulent M1_UK_ Clone, Canada, 2018–2023

**DOI:** 10.3201/eid3011.241068

**Published:** 2024-11

**Authors:** Alyssa R. Golden, Averil Griffith, Gregory J. Tyrrell, Julianne V. Kus, Allison McGeer, Marc-Christian Domingo, Jennifer Grant, Jessica Minion, Paul Van Caeseele, Guillaume Desnoyers, David Haldane, Yang Yu, Xiaofeng Ding, Laura Steven, Jan McFadzen, Courtney Primeau, Irene Martin

**Affiliations:** Public Health Agency of Canada, Winnipeg, Manitoba, Canada (A. Golden, A. Griffith, I. Martin); Provincial Laboratory for Public Health (Microbiology), Edmonton, Alberta, Canada (G.J. Tyrrell); Public Health Ontario, Toronto, Ontario, Canada (J.V. Kus); University of Toronto, Toronto (J.V. Kus); Mount Sinai Hospital, Toronto (A. McGeer); Institut National de Santé Publique du Québec, Sainte-Anne-de-Bellevue, Québec, Canada (M-.C. Domingo); British Columbia Centre for Disease Control, Vancouver, British Columbia, Canada (J. Grant); Roy Romanow Provincial Laboratory, Regina, Saskatchewan, Canada (J. Minion); Cadham Provincial Laboratory, Winnipeg (P. Van Caeseele); Laboratoire de Santé Publique du New Brunswick, Moncton, New Brunswick, Canada (G. Desnoyers); Queen Elizabeth II Health Science Centre, Halifax, Nova Scotia, Canada (D. Haldane); Newfoundland and Labrador Public Health Laboratory, St. John’s, Newfoundland, Canada (Y. Yu); Queen Elizabeth Hospital, Charlottetown, Prince Edward Island, Canada (X. Ding); Stanton Territorial Hospital Laboratory, Yellowknife, Northwest Territories, Canada (L. Steven); Yukon Communicable Disease Control, Whitehorse, Yukon, Canada (J. McFadzen); Public Health Agency of Canada, Ottawa, Ontario, Canada (C. Primeau)

**Keywords:** *Streptococcus pyogenes*, group A Streptococcus, *emm*1, M1_UK_, bacteria, antimicrobial resistance, scarlet fever, Canada

## Abstract

To determine invasive group A *Streptococcus* trends in Canada, we characterized *emm*1 isolates collected during 2018–2023. The percentage of hypervirulent M1_UK_ lineage isolates increased significantly, from 22.1% in 2018 to 60.2% in 2023. Genomic analysis identified geographically and temporally associated clusters and genes associated with virulent bacteriophage acquisition.

The hypervirulent M1_UK_ lineage of group A *Streptococcus* (GAS), originally identified in the United Kingdom in 2019, has been associated with increased notifications of scarlet fever and invasive GAS (iGAS) infections (*1*). The M1_UK_ lineage is characterized by increased production of *speA* (streptococcal pyrogenic exotoxin A) and is differentiated from the M1_global_ lineage by 27 key single-nucleotide variants (SNVs) (*1*). Initial characterization of a subset of *emm*1 isolates collected in Canada during 2016–2019 identified 10% of isolates as the M1_UK_ lineage (*2*).

Beginning in 2022, several health organizations, including the World Health Organization and the Pan American Health Organization, reported increased cases of pediatric iGAS in numerous member countries, above seasonal expectations (*3*,*4*). Many countries in Europe, including Belgium, Netherlands, and the United Kingdom (*5*–*7*), have associated increased iGAS disease in the 2022–23 season with *emm*1, particularly the M1_UK_ variant. In light of the worldwide increased iGAS disease activity and the association of those increases with *emm*1, we sought to describe the trends in *emm*1 and M1_UK_ in Canada during 2018–2023.

## The Study

We identified 2,582 isolates of iGAS *emm*1 collected during 2018–2023 as part of the passive, laboratory-based surveillance system for iGAS in Canada (*8*). Of those, we sequenced 2,315 isolates by using Illumina NextSeq technology (https://www.illumina.com); the remainder were received as line-listed typing data only. We identified M1_UK_ isolates by mapping whole-genome sequencing reads to reference strain MGAS5005 and identifying 27 characteristic SNVs, as previously described (*1*). We performed core SNV phylogenetic analysis by using the SNVPhyl pipeline (*9*) and identified genomic clusters by using ClusterPicker with default settings (*10*). We assessed presence of antimicrobial resistance, toxin, and virulence genes by using the WADE pipeline (https://github.com/phac-nml/wade), the public virulence factor database (http://www.mgc.ac.cn/VFs), and custom database queries. The M1_UK_ genomic data reported in our study have been deposited in the National Center for Biotechnology Information Sequence Read Archive (BioProject PRJNA1137869).

We assessed trends in lineage distribution for statistical significance by using the Cochran-Armitage test for trend and differences between lineages by using the 2-tailed Fisher exact test (α = 0.05). We aggregated data regionally into the Western (British Columbia, Alberta), Prairie (Saskatchewan, Manitoba), Central (Ontario, Québec), Eastern (New Brunswick, Nova Scotia, Prince Edward Island, Newfoundland and Labrador), and Northern (Yukon, Northwest Territories, Nunavut) regions of Canada.

**Figure 1:**
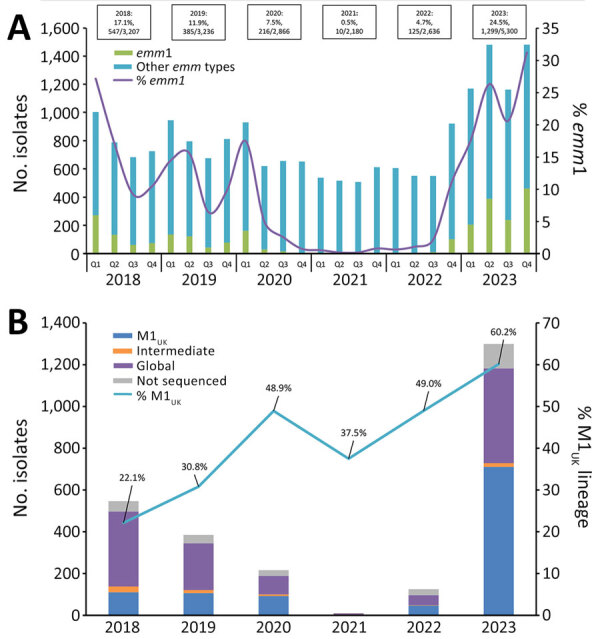
Expansion of invasive group A *Streptococcus* *emm*1 and the M1_UK_ lineage in Canada, 2018–2023. A) Number of *emm*1 isolates collected, by quarter. B) Percentage of M1_UK_ isolates among *emm*1 isolates collected. Q1, January–March; Q2, April–June; Q3, July–September; Q4, October–December. Annual proportions of *emm*1 are listed above the bars. Intermediate indicates an isolate with a partial M1_UK_ genotype; not sequenced indicates an isolate that was submitted to the National Microbiology Laboratory as line-listed typing data only.

In 2018, *emm*1 accounted for 17.1% of iGAS isolates collected in Canada, after which the proportion of *emm*1 decreased significantly, to a low of 0.5% in 2021 (p<0.0001), followed by a sharp increase to 24.5% in 2023 (p<0.0001) (Figure 1, panel A). Overall, during 2018–2023, a total of 46.2% of the 2,315 sequenced *emm*1 isolates were the M1_UK_ lineage. The proportion of M1_UK_ isolates increased from 22.1% (110/497) in 2018 to 60.2% (711/1,182) in 2023 (p<0.0001) (Figure 1, panel B). In 2023, the proportion of M1_UK_ was highest in the Prairie region (66.7%), followed by the Central (62.5%), Western (58.9%) and Eastern regions (35.3%); no M1_UK_ was collected in the Northern region. The only common (n>20) *emm*1 subtype associated exclusively with the M1_UK_ lineage was *emm*1.147; subtypes *emm*1.146 and *emm*1.25 were exclusively associated with the M1_global_ genotype. Subtypes *emm*1.0 and *emm*1.3 were associated with both M1_global_ and M1_UK_ genotypes (40.8% of *emm*1.0 and 95.0% of *emm*1.3 were M1_UK_).

Few antimicrobial resistance determinants were identified within the M1_global_ or M1_UK_ cohorts (Table). Compared with other *emm*1 isolates, the M1_UK_ variant demonstrated significantly higher presence of genes *speC* (streptococcal pyrogenic exotoxin C) and *ssa* (streptococcal superantigen), as well as virulence factors *spd1* (phage-associated DNase) and *hylP* (phage-associated hyaluronidase).

**Table:** Characteristics of M1_UK_ lineage and other invasive group A *Streptococcus* *emm*1 isolates collected in Canada, 2018–2023

Isolate feature*	Lineage, no. (%) isolates		p value‡
	M1_UK_, n = 1,069	Other *emm*1, n = 1,246†	
Antimicrobial susceptibility			
Penicillin	100	100	1.000
Erythromycin	99.5 (1,064)	99.2 (1,236)	0.4375
Clindamycin	99.8 (1,067)	99.5 (1,240)	0.2997
Chloramphenicol	100	100	1.000
Levofloxacin	99.9 (1,068)	99.8 (1,243)	0.6291
Tetracycline	99.6 (1,065)	99.1 (1,235)	0.1928
Toxin gene presence			
* speA*	98.4 (1,052)	97.4 (1,214)	0.1124
* speC*	43.8 (468)	8.3 (103)	<0.0001
* speG*	100 (1,069)	99.9 (1,245)	1.000
* speH*	0	0	1.000
* speI*	0	0	1.000
* speJ*	99.5 (1,064)	99.4 (1,238)	0.7817
* speK*	0.1 (1)	0	0.4618
* speL*	0	0	1.000
* speM*	0	0	1.000
* smeZ*	98.4 (1,052)	98.9 (1,232)	0.3674
* ssa*	35.5 (379)	4.4 (55)	<0.0001
Virulence gene presence			
Phage-associated DNase, *spd1*	43.9 (469)	8.3 (103)	<0.0001
Phage-associated hyaluronidase, *hylP*	37.8 (404)	3.9 (49)	<0.0001
Gene combinations			
* speC* + *ssa* + *spd1*	35.4 (378)	8.3 (55)	<0.0001
* speC* + *spd1*	8.3 (89)	3.9 (48)	<0.0001

*All isolate features (antimicrobial susceptibilities, toxin, and virulence gene presence) were inferred from whole-genome sequences. †Other *emm*1 with whole-genome sequencing data available. ‡Two-tailed; p<0.05 considered significant.

Phylogenetic analysis of all *emm*1 isolates identified clear separation of the M1_global_ and M1_UK_ lineages (Appendix Figure 1); the isolates within the M1_UK_ cluster differed from those in the M1_global_ cluster by an average of 46 (range 22–80) SNVs. Within the M1_UK_ cluster, isolates differed by an average of 17.6 (range 0–41) SNVs, and there was more variability within isolates of the M1_global_ lineage (average 32.6 [range 0–82] SNVs difference).

**Figure 2:**
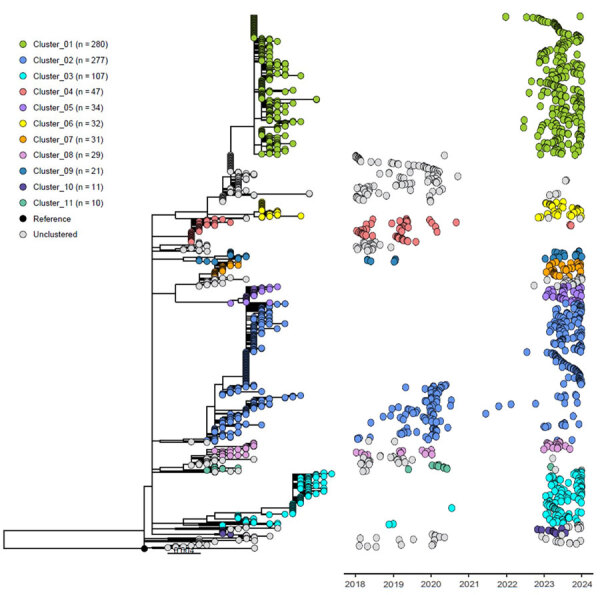
Maximum-likelihood core single-nucleotide variant phylogeny for 1,069 invasive group A *Streptococcus* M1_UK_ lineage isolates collected in Canada, 2018–2023. Eleven large clusters are shown, each containing 10–280 isolates.

ClusterPicker identified 11 large clusters within the M1_UK_ cohort, each cluster containing 10–280 isolates (Figure 2; Appendix Table). In general, the highest proportion of each cluster was collected in 2023, which is consistent with the surge of iGAS disease cases that year. Exceptions include clusters 4 and 11, which included isolates predominantly collected before the *emm*1 decrease that coincided with the COVID-19 pandemic. Clusters 1, 5–7, and 10 were identified exclusively after the COVID-19 pandemic period, and clusters 2 and 8 persisted across the study period. Clusters were generally associated with geographic region: clusters 1, 2, 5, 7, and 10 were strongly associated with the Central region, and clusters 3, 6, 8, and 9 were strongly associated with associated with the Western region. Cluster 4 was mostly found in the Eastern region; Cluster 11 was predominantly from the Prairie region. Within the Central region, approximately two thirds of the total isolates collected during the study period were part of either cluster 1 or cluster 2. Cluster 3 was most common for isolates collected from the Western (40.7%) and Prairie (25.0%) regions, and cluster 4 was most common in the Eastern region (53.8%).

More than 99% of isolates within M1_UK_ cluster 1 possessed *speC*, *ssa*, *spd,1*, and *hylP* (Appendix Table). Within cluster 3, a total of 78.5% of isolates possessed *speC* and *spd1*; presence of *ssa* and *hylP* was sporadic. Those 4 genes were sporadically present within clusters 2 and 7.

## Conclusions

Our study highlights expansion of the M1_UK_ GAS lineage in Canada. Initial genomic characterization of *emm*1 isolates in Canada identified only 10% M1_UK_ in a subset of *emm*1 isolates collected during 2016–2019 (*2*); by 2023, M1_UK_ comprised 60.2% of *emm*1 isolates. The proportion of M1_UK_ in Canada in 2023 is much higher than that most recently published from the United States (11%), although considerably lower than that reported by recent studies from Belgium (78%) and the United Kingdom (95.7%) (*5*,*7*,*11*). Our study findings are consistent with findings of Vieira et al., who noted that the M1_UK_ lineage showed less genomic diversity than the M1_global_ lineage (*7*). Of note, we did not identify any isolates of the novel M1_DK_ lineage, which was originally identified in Denmark and was responsible for 30% of iGAS cases in Denmark in winter 2022–23 (*12*).

Our study identified a large proportion of M1_UK_ isolates with the bacteriophage-encoded DNase *spd1* and *speC*/*ssa* superantigens. The presence of those 3 genes suggests acquisition of a virulent prophage related to ΦHKU488.vir, which has been associated with outbreaks of *emm*12 scarlet fever in Asia (*13*,*14*). However, the lack of antimicrobial resistance determinants in Canadian *emm*1 isolates so far indicates limited transfer of the integrative conjugative elements that have been responsible for macrolide, lincosamide, and tetracycline resistance in GAS outbreaks in Asia (*14*). Although M1_UK_ with this phage were present in Canada before the COVID-19 pandemic, their presence substantially expanded in 2023, particularly in central Canada (Figure 2, cluster 1). M1_UK_ isolates within cluster 3 were associated with *speC* and *spd1* only; that combination is associated with a different prophage, ΦSP370.1 (*15*). Those isolates were more common in western Canada beginning in 2023, suggesting a different path of virulence gene acquisition compared with that of ΦHKU488.vir. Monitoring the spread of the variants of M1_UK_, particularly for development of antimicrobial resistance, will remain critical. Our study underscores the value of linking laboratory data to epidemiologic variables to enhance our knowledge of how GAS variants affect clinical manifestations, outcomes, and risk groups.

Appendix Additional information for study of invasive group A *Streptococcus* hypervirulent M1_UK_ clone, Canada, 2018–2023.

## References

[1] Lynskey NN, Jauneikaite E, Li HK, Zhi X, Turner CE, Mosavie M, et al. Emergence of dominant toxigenic M1T1 *Streptococcus pyogenes* clone during increased scarlet fever activity in England: a population-based molecular epidemiological study. Lancet Infect Dis. 2019;19:1209–18. 10.1016/S1473-3099(19)30446-3 31519541 PMC6838661

[2] Demczuk W, Martin I, Domingo FR, MacDonald D, Mulvey MR. Identification of Streptococcus pyogenes M1_UK_ clone in Canada. Lancet Infect Dis. 2019;19:1284–5. 10.1016/S1473-3099(19)30622-X 31782392

[3] World Health Organization. Disease outbreak news: increased incidence of scarlet fever and invasive group A *Streptococcus* infection—multi-country [cited 2023 Nov 26]. https://www.who.int/emergencies/disease-outbreak-news/item/2022-DON429

[4] Pan American Health Organization/World Health Organization. Informative note: cases of diseases caused by group A *Streptococcus* in Uruguay [in Spanish] [cited 2023 Nov 28]. https://www.paho.org/es/documentos/nota-informativa-casos-enfermedades-causadas-por-estreptococo-grupo-uruguay

[5] Rodriguez-Ruiz JP, Lin Q, Lammens C, Smeesters PR, van Kleef-van Koeveringe S, Matheeussen V, et al. Increase in bloodstream infections caused by *emm*1 group A *Streptococcus* correlates with emergence of toxigenic M1_UK_, Belgium, May 2022 to August 2023. Euro Surveill. 2023;28:2300422. 10.2807/1560-7917.ES.2023.28.36.2300422 37676145 PMC10486196

[6] van der Putten BCL, Vlaminckx BJM, de Gier B, Freudenburg-de Graaf W, van Sorge NM. Group A streptococcal meningitis with the M1_UK_ variant in the Netherlands. JAMA. 2023;329:1791–2. 10.1001/jama.2023.5927 37027150 PMC10082416

[7] Vieira A, Wan Y, Ryan Y, Li HK, Guy RL, Papangeli M, et al. Rapid expansion and international spread of M1_UK_ in the post-pandemic UK upsurge of Streptococcus pyogenes. Nat Commun. 2024;15:3916. 10.1038/s41467-024-47929-7 38729927 PMC11087535

[8] Golden AR, Griffith A, Tyrrell GJ, Kus JV, McGeer A, Domingo MC, et al. Invasive group A streptococcal disease surveillance in Canada, 2021-2022. Can Commun Dis Rep. 2024;50:135–43. 10.14745/ccdr.v50i05a03 38835501 PMC11149783

[9] Petkau A, Mabon P, Sieffert C, Knox NC, Cabral J, Iskander M, et al. SNVPhyl: a single nucleotide variant phylogenomics pipeline for microbial genomic epidemiology. Microb Genom. 2017;3:e000116. 10.1099/mgen.0.000116 29026651 PMC5628696

[10] Ragonnet-Cronin M, Hodcroft E, Hué S, Fearnhill E, Delpech V, Brown AJ, et al.; UK HIV Drug Resistance Database. Automated analysis of phylogenetic clusters. BMC Bioinformatics. 2013;14:317. 10.1186/1471-2105-14-317 24191891 PMC4228337

[11] Li Y, Rivers J, Mathis S, Li Z, Chochua S, Metcalf BJ, et al. Expansion of Invasive Group A Streptococcus M1_UK_ Lineage in Active Bacterial Core Surveillance, United States, 2019‒2021. Emerg Infect Dis. 2023;29:2116–20. 10.3201/eid2910.230675 37640370 PMC10521608

[12] Johannesen TB, Munkstrup C, Edslev SM, Baig S, Nielsen S, Funk T, et al. Increase in invasive group A streptococcal infections and emergence of novel, rapidly expanding sub-lineage of the virulent *Streptococcus pyogenes* M1 clone, Denmark, 2023. Euro Surveill. 2023;28:2300291. 10.2807/1560-7917.ES.2023.28.26.2300291 37382884 PMC10311951

[13] Davies MR, Keller N, Brouwer S, Jespersen MG, Cork AJ, Hayes AJ, et al. Detection of Streptococcus pyogenes M1_UK_ in Australia and characterization of the mutation driving enhanced expression of superantigen SpeA. Nat Commun. 2023;14:1051. 10.1038/s41467-023-36717-4 36828918 PMC9951164

[14] Ben Zakour NL, Davies MR, You Y, Chen JHK, Forde BM, Stanton-Cook M, et al. Transfer of scarlet fever-associated elements into the group A *Streptococcus* M1T1 clone. Sci Rep. 2015;5:15877. 10.1038/srep15877 26522788 PMC4629146

[15] McShan WM, McCullor KA, Nguyen SV. The bacteriophages of *Streptococcus pyogenes.* Microbiol Spectr. 2019;7:7.3.8. 10.1128/microbiolspec.gpp3-0059-2018 PMC11314938 31111820

