# Supplementary material for: Invasive Group A Streptococcus Hypervirulent M1UK Clone, Canada, 2018–2023
Source: Emerg Infect Dis. 2024 Nov;30(11):2409–13. doi: 10.3201/eid3011.241068 (PMC11521172; doi:10.3201/eid3011.241068)
Supplement: Appendix — Additional information for study of invasive group A Streptococcus hypervirulent M1UK clone, Canada, 2018–2023. [file 24-1068-Techapp-s1.pdf]

Article DOI: https://doi.org/10.3201/eid3011.241068

EID cannot ensure accessibility for supplementary materials supplied by authors. Readers who have difficulty accessing supplementary content should contact the authors for assistance.

Invasive Group A *Streptococcus* Hypervirulent M1$_{UK}$ Clone, Canada, 2018–2023

Appendix

Appendix Table. Summary of temporal, geographic and virulence characteristics of eleven M1$_{UK}$ clusters in Canada

		Year, % of cluster (% of yearly M1$_{UK}$ [b])						Canadian Region [c], % of cluster (% of regional M1$_{UK}$ [b])					Avg SNV	Gene presence, % of cluster			
Cluster	N	2018	2019	2020	2021	2022	2023	Western	Prairie	Central	Eastern	*emm*1 subtypes within cluster (n)	difference within cluster (range)	speC	ssa	spd1	hylP
1	280	-	-	-	0.4 (33.3)	12.9 (76.6)	86.8 (34.2)	1.1 (2.0)	-	95.4 (34.3)	3.6 (12.5)	1.0 (2), 1.112 (1), 1.147 (59), 1.3 (216), NT (2)	3.3 (0-14)	99.3	99.6	100	100
2	277	2.9 (7.3)	11.6 (30.2)	17.7 (53.3)	0.7 (66.7)	1.4 (8.5)	65.7 (25.6)	1.1 (2.0)	2.9 (13.3)	92.4 (32.9)	3.6 (12.5)	1.0 (277)	5.7 (0-20)	2.5	1.8	2.2	4.3
3	107	1.9 (1.8)	-	0.9 (1.1)	-	-	97.2 (14.6)	57.0 (40.7)	14.0 (25.0)	18.7 (2.6)	10.3 (13.8)	1.0 (105), 1.49 (1), 1.6 (1)	9.4 (0-31)	78.5	1.9	78.5	1.9
4	47	34.0 (14.5)	53.2 (23.6)	8.5 (4.3)	-	-	4.3 (0.3)	8.5 (2.7)	-	-	91.5 (53.8)	1.0 (47)	1.9 (0-9)	-	-	-	-
5	34	-	-	-	-	-	100 (4.8)	2.9 (0.7)	-	97.1 (4.2)	-	1.0 (34)	2.1 (0-9)	-	-	-	-
6	32	-	-	-	-	6.3 (4.3)	93.8 (4.2)	37.5 (8.0)	31.3 (16.7)	31.3 (1.3)	-	1.3 (32)	1.9 (0-9)	-	-	-	-
7	31	-	-	-	-	-	100 (4.4)	-	-	100 (4.0)	-	1.0 (31)	1.8 (0-6)	3.2	3.2	3.2	3.2
8	29	17.2 (4.5)	20.7 (5.7)	6.9 (2.2)	-	3.4 (2.1)	51.7 (2.1)	82.8 (16.0)	6.9 (3.3)	10.3 (0.4)	-	1.0 (29)	5.5 (0-11)	-	-	-	-
9	21	14.3 (2.7)	14.3 (2.8)	-	-	-	71.4 (2.1)	66.7 (9.3)	-	33.3 (0.9)	-	1.0 (21)	4.7 (0-12)	-	-	-	-
10	11	-	-	-	-	18.2 (4.3)	81.8 (1.3)	9.1 (0.7)	-	90.9 (1.3)	-	1.0 (11)	1.2 (0-6)	-	-	-	9.1
11	10	-	10.0 (0.9)	80.0 (8.7)	-	-	10.0 (0.1)	10.0 (0.7)	90.0 (15.0)	-	-	1.0 (9), 1.74 (1)	2.2 (0-6)	-	-	-	-
UC	190	40.0 (69.1)	20.5 (36.8)	14.7 (30.4)	-	1.1 (4.3)	23.7 (6.3)	13.7 (17.3)	8.4 (26.7)	74.7 (18.2)	3.2 (7.5)	1.0 (95), 1.3 (95)	-	51.6	48.4	51.6	48.9
Total	1,069	110	106	92	3	47	711	150	60	779	80	-	-	468	379	469	389

[a]Clusters identified by ClusterPicker, see Figure 2. [b] Yearly M1$_{UK}$ with WGS data available; [c] Western, British Columbia and Alberta; Prairie, Saskatchewan and Manitoba; Central, Ontario and Quebec; Eastern, Newfoundland and Labrador, Nova Scotia, New Brunswick, Prince Edward Island; no M1$_{UK}$ isolates collected from Northern Canada during the study period. UC, unclustered.

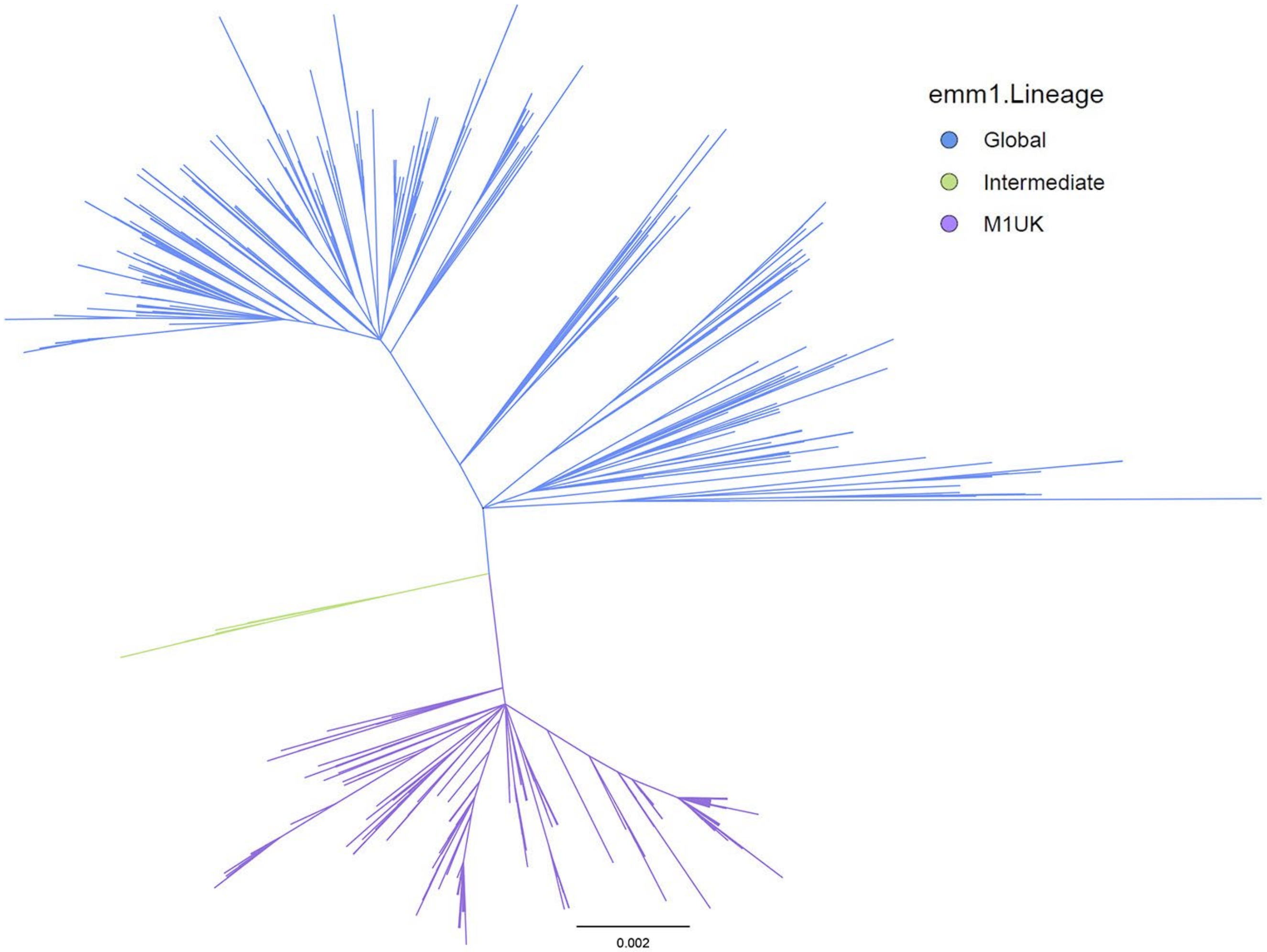

Appendix Figure. Maximum likelihood core SNV phylogeny for 2,315 *emm*1 isolates collected in Canada, 2018 to 2023.

[a]Intermediate refers to an isolate with a partial $M1_{UK}$ genotype.
